# Explainable Machine Learning Framework for Dynamic Monitoring of Disease Prognostic Risk: Retrospective Cohort Study

**DOI:** 10.2196/65585

**Published:** 2025-08-07

**Authors:** Tetsuo Ishikawa, Masahiro Shinoda, Megumi Oya, Koichi Ashizaki, Shinichiro Ota, Kenichi Kamachi, Kazuhiro Sakurada, Eiryo Kawakami, Masaharu Shinkai

**Affiliations:** 1 Predictive Medicine Special Project, RIKEN Center for Integrative Medical Sciences, RIKEN, 1-7-22 Suehiro-cho, Tsurumi-ku, Yokohama, 230-0045, Japan, 81 45-503-7000; 2 Department of Extended Intelligence for Medicine, The Ishii-Ishibashi Laboratory, Keio University School of Medicine, Tokyo, Japan; 3 Department of Artificial Intelligence Medicine, Graduate School of Medicine, Chiba University, Chiba, Japan; 4 Department of Respiratory Medicine, Tokyo Shinagawa Hospital, Tokyo, Japan; 5 Collective Intelligence Research Laboratory, Graduate School of Arts and Sciences, The University of Tokyo, Tokyo, Japan; 6 Division of Applied Mathematical Science, RIKEN Center for Interdisciplinary Theoretical and Mathematical Sciences, RIKEN, Yokohama, Japan

**Keywords:** COVID-19, prognostic modeling, electronic health records, time-varying biomarkers, clinical decision support, interpretable AI, random survival forests

## Abstract

**Background:**

Patients’ clinical status often evolves rapidly after an initial diagnosis, with each patient exhibiting a distinct disease trajectory. As a result, static risk scores fall short in supporting timely interventions—an issue highlighted by COVID-19, where deaths have stemmed from heterogeneous pathways such as pneumonia, multiorgan failure, or exacerbation of preexisting conditions.

**Objective:**

This study aims to propose a dynamic prognostic risk assessment framework based on longitudinal data collected during hospitalization, using COVID-19 as an example. Our aim was to develop and validate an interpretable framework that (1) screens prognosis at admission and (2) dynamically updates mortality risk throughout hospitalization, thereby providing clinicians with early, explainable warnings while minimizing additional cognitive load.

**Methods:**

In this retrospective study, we extracted electronic medical records of 382 COVID-19 cases treated at Tokyo Shinagawa Hospital between January 27 and September 30, 2020. At admission, gradient boosting decision trees (Light Gradient Boosting Machine) were used to predict the maximum clinical deterioration, including death, based on data available at initial diagnosis. Model performance was evaluated using the area under the receiver operating characteristic curve (AUC). For in-hospital monitoring, random survival forests (RSF) were trained on a longitudinal dataset that combined static demographic characteristics with serially measured vital signs and laboratory results. The model dynamically assessed daily mortality risk by calculating a 7-day cumulative hazard function, with risk scores recalculated each day during hospitalization. RSF accuracy was evaluated in an independent one-third test set using the concordance index (C-index), an integrated Brier score (1-50 days), and mean time-dependent AUC. SurvSHAP(t), an extension of Shapley Additive Explanations, was applied to provide time-dependent explanations of each variable’s contribution to the prediction.

**Results:**

The prediction at initial diagnosis showed good agreement with the actual severity outcomes (AUC of 0.717 for predicting hospitalization/severity ≥2; 0.878 for severity ≥3; 0.951 for severity ≥4; 0.952 for severity ≥5; and 0.970 for death/severity=6), although some cases exhibited discrepancies between the predicted and actual prognoses. The dynamic mortality risk assessment during hospitalization using the RSF achieved a test-set C-index of 0.941, an integrated Brier score of 0.315, and a mean time-dependent AUC of 0.936. This dynamic assessment was able to distinguish between dead and surviving patients as early as 1‐2 weeks before the outcome. Early in hospitalization, C-reactive protein was an important risk factor for mortality; during the middle period, peripheral oxygen saturation (SpO_2_) gained importance; and immediately before death, platelets and β-D-glucan were the primary risk factors.

**Conclusions:**

Integrating static admission triage with daily, explainable RSF predictions enables early identification of patients with COVID-19 at high risk of deterioration. By surfacing phase-specific, actionable predictors, the framework supports timely interventions and more efficient resource allocation. Prospective, multicenter studies are warranted to validate its generalizability and clinical impact.

## Introduction

During hospitalization, various adverse events and complications arise from the disease and its treatment, sometimes leading to death [1]. Therefore, health care professionals take great care not to overlook signs of deterioration based on blood tests and vital-sign measurements [2,3]. The recent surge in hospital admissions driven by COVID-19 and population aging has intensified this burden [4,5]. Predicting patient outcomes and issuing timely alerts can reduce the burden and enable the advanced preparation of scarce medical resources. To frame the scope of this study, we ask a single overarching question: How can clinicians anticipate deterioration early enough to intervene, while minimizing additional cognitive load?

The task is complicated by the heterogeneity of risk factors and signs of deterioration, which makes uniform risk assessment challenging, even for patients with the same disease [6]. Taking COVID-19 as an example, causes of death range from pneumonia to multiorgan failure and exacerbation of preexisting diseases [7]. Detecting such diverse paths to deterioration requires a personalized monitoring strategy that tracks multiple prognostic biomarkers over time. This clinical heterogeneity defines the modeling gap that data-driven methods must fill.

Recent reports indicate that machine learning models using electronic health records can accurately predict individual deterioration and mortality, thereby aiding health care professionals in clinical decision-making [8,9]. However, most existing prognostic models are trained on static snapshots, struggle to generalize across heterogeneous populations, or fail to provide interpretable insights. While deep learning models applied to longitudinal intensive care unit data can assess risks dynamically [10,11], their “black-box” nature restricts bedside adoption [12]. Accordingly, the next generation of prognostic tools must be both dynamic and interpretable to earn clinical trust.

Real-world deployment adds further hurdles: missing data, irregular sampling, inconsistent documentation, and limited computation resources in busy wards. Methods must therefore be accurate, explainable, and robust to data imperfections, while remaining fast enough for routine use. Taken together, limited interpretability, data-quality issues, and computational constraints form a 3-fold barrier that this work seeks to overcome.

To address these limitations, we leverage random survival forests (RSFs) to capture time-varying trajectories and pair them with SurvSHAP(t), an extension of Shapley Additive Explanations (SHAP) that provides context-specific, time-dependent rationale [13]. In contrast to models that assume linear relationships (eg, Cox proportional hazards), RSF flexibly models nonlinear, time-dependent interactions; SurvSHAP(t) embeds explanation directly alongside prediction, closing the trust gap.

**Figure 1.**
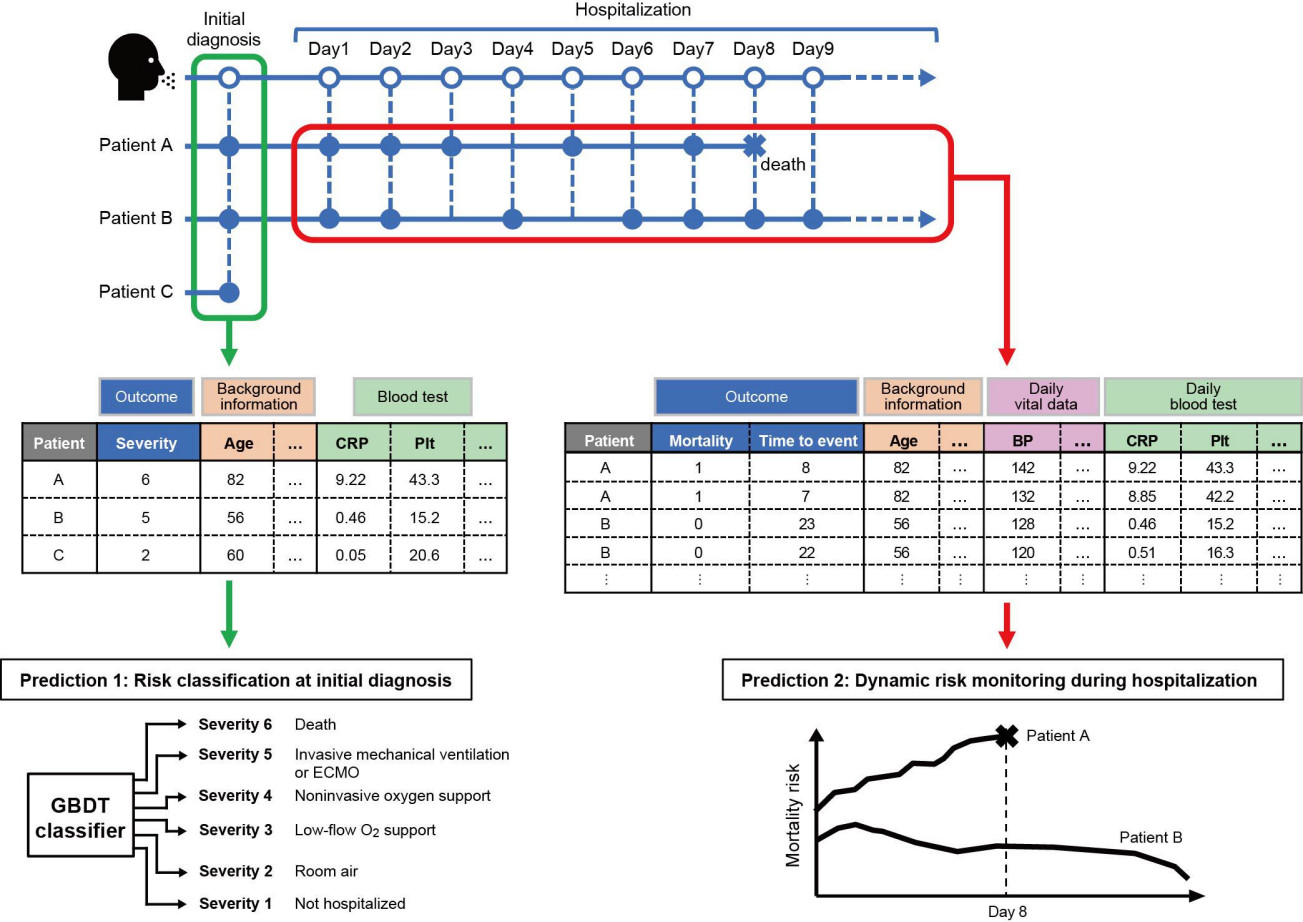
Conceptual diagram illustrating the diversity of pathological changes. In this study, we first assess the ability to predict a patient’s maximum COVID-19 severity using data from the initial diagnosis. We then introduce a dynamic prognostic risk assessment framework that continuously monitors hospitalized patients based on their longitudinal data. BP: blood pressure; CRP: C-reactive protein; ECMO: extracorporeal membrane oxygenation; GBDT: gradient boosting decision trees; Plt: platelet.

Building on these insights, we first confirm the predictability of prognosis at initial diagnosis with a machine learning model that anticipates the highest COVID-19 severity from baseline data. We then propose a dynamic prognostic-risk framework that continuously updates survival estimates using longitudinal information (Figure 1) and visualizes the drivers of individual risk. This 2-stage design—static triage at admission followed by dynamic risk updates—constitutes the narrative arc of the paper.

We therefore present an interpretable, dynamic prognostic framework designed to enhance clinicians’ understanding of patient status, enable precise treatment allocation, and optimize the use of medical resources.

## Methods

### Study Design, Setting, and Population

This retrospective cohort study screened 382 patients with COVID-19 treated between January 2020 and September 2020 at the Department of Respiratory Medicine, Tokyo Shinagawa Hospital. Patients aged under 18 years and those with unknown severity changes during hospitalization or clinical outcomes were excluded from the analysis. This included cases in which the prognosis was unclear because of hospital transfer, observation censoring, or insufficient documentation of the time point at which severity changed. Inpatients with a discrepancy of more than 4 days between the date of first diagnosis and the date of admission were excluded from the prognostic screening but were included in the dynamic risk assessment. The included cases were randomly divided into training and test datasets at a 2:1 ratio using a stratified sampling method to preserve the distribution of severity outcomes.

For risk classification at initial diagnosis, patients who experienced severity changes were included in the dataset. Overall, 201 outpatients and 161 inpatients were included. We extracted data from the electronic medical records, including 84 variables, such as symptoms, background information, and blood and urine biomarkers, for prognostic screening at initial diagnosis (Multimedia Appendix 1).

For the dynamic prognostic risk assessment framework, the dataset included 182 inpatients with longitudinal data collected during hospitalization. From the electronic medical records, 72 variables, including blood and urine biomarkers, vital signs, and background information during hospitalization, were extracted for dynamic risk assessment (Multimedia Appendix 2). The median length of stay for inpatients was 11 days.

### Ethical Considerations

This study was approved by the local institutional review board of RIKEN and Tokyo Shinagawa Hospital (approval number 20-A-06). It was conducted in accordance with the 1964 Declaration of Helsinki and its later amendments, or comparable ethical standards. The requirement for informed consent was waived because of the retrospective nature of the study. All data used in this study were deidentified before analysis. Specifically, any directly identifiable personal information (eg, names, addresses, or medical record numbers) was removed. The deidentification process adhered to relevant institutional and national data protection guidelines. As the data were not anonymized in the strictest sense, but were instead deidentified with safeguards in place, individual participants cannot be readily identified by the investigators.

### Severity Classification

We adopted the oxygen-support status scores proposed by Grein et al [14] to classify patient severity. The score uses an ordinal scale from 1 to 6 based on the level of oxygenation: (1) discharged or not hospitalized; (2) room air; (3) low-flow oxygen support; (4) noninvasive intervention, including nasal high-flow oxygen therapy or noninvasive positive pressure ventilation; (5) invasive mechanical ventilation or extracorporeal membrane oxygenation (ECMO); and (6) death. In addition to the original score, where 1 indicated discharge status, outpatients were assigned a score of 1.

### Machine Learning for Prognostic Classification at Initial Diagnosis

Gradient boosting decision trees (GBDT) [15,16] were used to predict the highest severity from data at initial diagnosis. GBDT is related to random forest and uses boosting to enhance predictive performance. Among various GBDT implementations, the Python package (Python Foundation) Light Gradient Boosting Machine (LightGBM; version 4.5.0) was used because of its superior computing speed. We included all variables with less than 15% missing values, given that COVID-19 severity and mortality are influenced by a wide range of factors. Although 265 biomarkers were recorded at the first visit, most were not measured in nonhospitalized patients. To ensure that the selected biomarkers would be applicable across a broad clinical spectrum, we included only laboratory test variables that were routinely measured in both hospitalized and nonhospitalized (outpatient) patients at the time of initial diagnosis. This approach was intended to support early-stage risk prediction and avoid biasing the model toward inpatient-specific features. Biomarkers available in less than 50% of the patient population were excluded to reduce the impact of missing data on model performance, resulting in 53 biomarkers collected from 4 days before to 1 day after the initial diagnosis. Missing values were imputed using the missForest package (version 1.5) in R (R Foundation) [17].

As a preprocessing step, variables with absolute Spearman correlation coefficients of 0.85 or higher in the training data were excluded, retaining only those with the strongest correlation with severity. While this univariate filtering approach does not account for potential multivariate interactions among features, it was adopted to reduce feature redundancy and multicollinearity, which could otherwise impair the stability and efficiency of model training.

LightGBM hyperparameters were optimized using Bayesian optimization with repeated cross-validation via Optuna version 4.2.1 [18]. We applied the best model to the test dataset to obtain predicted probabilities and plotted the corresponding receiver operating characteristic curve. The area under the receiver operating characteristic curve was calculated to evaluate the predictive performance of the initial diagnosis model for each severity classification. To evaluate the importance of variables in the prediction, we used SHAP values [13] for the test data. Importantly, when models were developed for different severity subgroups, hyperparameter optimization and SHAP analysis were performed separately for each group to account for subgroup-specific characteristics.

To validate the advantage of the selected LightGBM model, we initially implemented and evaluated 5 machine learning algorithms for predicting hospital admission (ie, threshold ≥2): LightGBM, random forest, support vector machine, Elastic Net, and decision tree. Among these, LightGBM and random forest demonstrated the highest predictive performance for binary classification (Figure S1 in Multimedia Appendix 3 and Table S3 in Multimedia Appendix 4). However, random forest required substantially longer training and inference times. Considering the need for computational efficiency and scalability in clinical applications, we selected LightGBM for subsequent analyses involving higher severity cut-offs.

### Dynamic Risk Assessment of Mortality During Hospitalization

We used survival models, including Cox proportional hazards models and RSFs [19], to evaluate patient mortality risk during hospitalization. The Cox proportional hazards model analyzes time-to-event data and associated predictors under the proportional hazards assumption. To accommodate a large number of predictors, regularized versions of the Cox model were used, including Cox-LASSO (least absolute shrinkage and selection operator) (L1), Cox-Ridge (L2), and Cox ElasticNet (a combination of L1 and L2 penalties).

To emulate real-time risk assessment, we implemented a dynamic updating mechanism in which the model recalculates cumulative hazard scores on each day of hospitalization. For each patient, the RSF model is applied iteratively at time points *t*, *t*+1, *t*+2, and so on, using only data observed up to the corresponding time. This time-restricted evaluation prevents data leakage from future observations and allows us to assess how well the model performs when used prospectively in a clinical setting. The RSF estimates the cumulative hazard function (CHF) by aggregating the outputs of multiple survival trees constructed using the random forest algorithm. Like random forest, RSF is robust to outliers and enables accurate risk assessment for event occurrence. To dynamically predict mortality risk, 72 variables were used in the analysis, including background factors (eg, age and BMI), as well as biomarkers and vital signs measured over time during hospitalization. We used variables that were measured in most patients (>180 patients for biomarkers and >150 patients for background and vital information). Missing values were imputed using the last observation carried forward method; if no prior values were available, continuous variables were filled with the median, and categorical variables with the mode. The Python (Python Foundation) package scikit-survival, which includes implementations of Cox and RSF models, was used for the analysis. To evaluate the dynamic risk of mortality, we used the 7-day CHF (ie, the estimated probability of death within 7 days) calculated using the RSF as an index of mortality risk. The 7-day CHF was calculated for each patient on each day of hospitalization.

The performance of the survival models was evaluated using 3 metrics: concordance index (C-index), integrated Brier score, and mean cumulative/dynamic AUC (mean AUC).

In clinical practice, it is important to identify the factors that increase the risk of mortality. The variable importance of the RSF models was calculated for the test data using SurvSHAP(t) [20], which provides a time-dependent explanation of the survival function predicted using SHAP. SurvSHAP(t) allowed us to determine how each variable affects the survival function at each time point. We obtained the mean aggregated SurvSHAP(t) across all days of hospitalization, which showed how each variable contributed to the prediction for every patient at every time point.

### Variable Selection for Dynamic Risk Assessment

To balance clinical applicability with the computational cost of SurvSHAP(t), we performed variable selection during the RSF training process. First, we trained an RSF model using all 72 variables on the training data and calculated permutation importance scores using the implementation in the scikit-learn Python package. The RSF model was then retrained using the top 10 variables with the highest permutation importance.

Additional technical details of the machine learning implementation not covered in the main text are presented in Multimedia Appendix 5.

## Results

### Patient Demographics

The median age of the 382 patients with COVID-19 was 39 years; 233 were male and 149 were female (Table 1). Of the 51 inpatients who required oxygen, 30 required low-flow oxygen, 3 needed high-flow oxygen, 8 were treated with invasive ventilation/ECMO, and 10 died.

**Table 1.** Baseline characteristics of patients with COVID-19 by severity outcome.

	Severity outcome of COVID-19					
	1: Not admitted	2: Room air	3: Oxygen administration	4: Noninvasive ventilation	5: ECMO^a^/invasive ventilation	6: Death
Number of cases, n	201	130	30	3	8	10
Length of stay (days), median (range)	N/A^b^	10 (2-52)	20 (11-36)	47 (31-48)	39 (25-73)	23 (12-37)
Age (years), median (range)	34 (18-93)	39 (19-98)	59 (30-93)	70 (68-85)	58 (49-75)	79 (50-94)
Sex, n (%)						
Female	75 (37)	60 (46)	9 (30)	2 (67)	2 (25)	1 (10)
Male	126 (63)	70 (54)	21 (70)	1 (33)	6 (75)	9 (90)

a ECMO: extracorporeal membrane oxygenation.

b N/A: not applicable.

### Prognostic Predictability of COVID-19 at Initial Diagnosis

**Figure 2.**
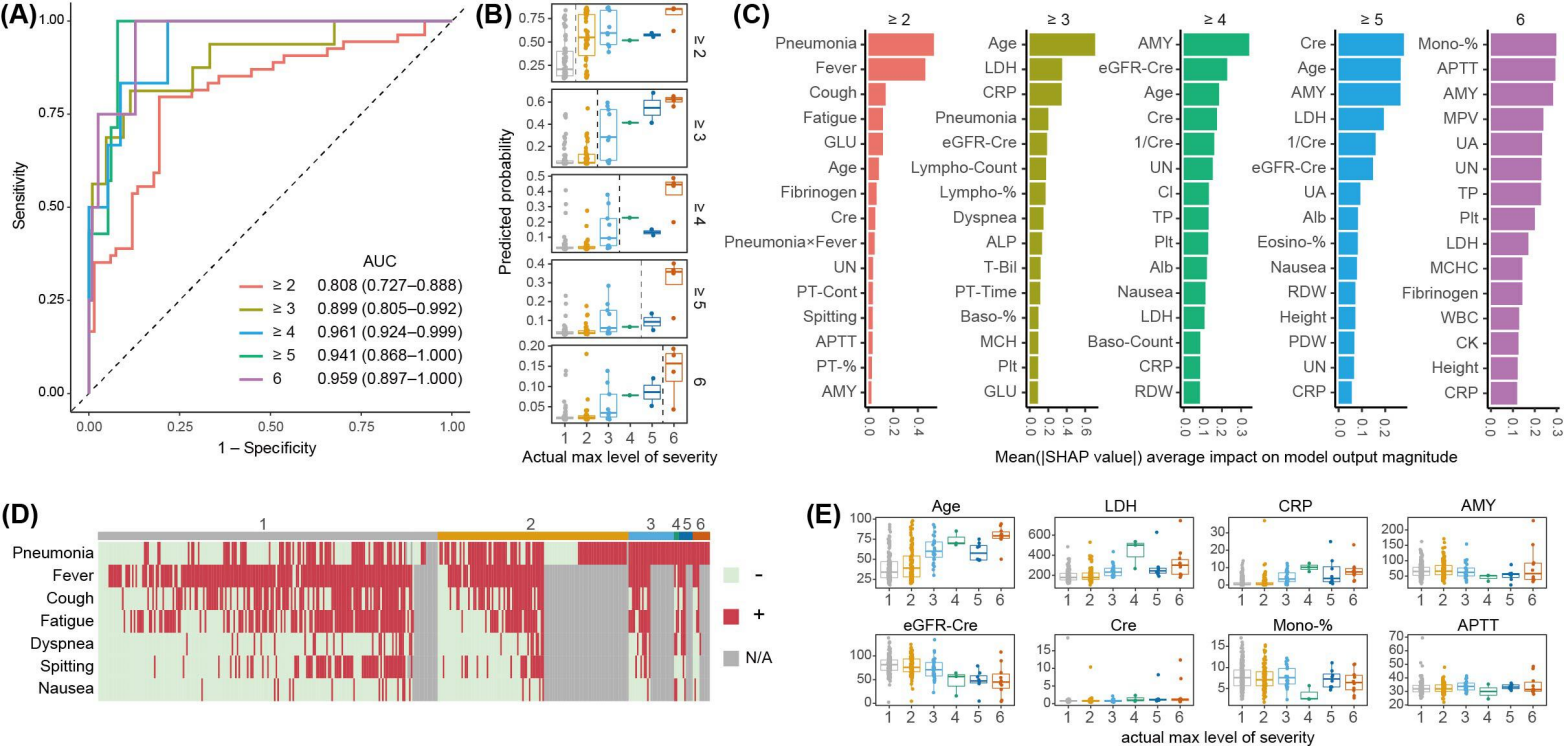
Screening for severity prognosis at the initial diagnosis. (A) Receiver operating characteristic curves and the AUC values for prediction models of severity outcomes based on the oxygen-support status score. The 95% CIs for the AUC are shown in parentheses. (B) Box and jitter plots showing the prediction probabilities that test cases exceed the severity threshold (indicated by the dashed line). The center line of the box indicates the median; box limits represent the upper and lower quartiles; whiskers extend to 1.5× the IQR. Each dot represents an individual observation. (C) Variable importance in each predictive model evaluated using SHAP values. Bar plots show the mean absolute SHAP value for each variable across individual patients in the test dataset. (D) Heatmap of symptoms observed at initial diagnosis. Columns represent individual patients, ordered by severity level and sorted to minimize missing values and symptom absence. (E) Box and jitter plots showing the distribution of key continuous variables across severity groups. Plot elements are defined as in panel B. Alb: albumin; ALP: alkaline phosphatase; AMY: amylase; APTT: activated partial thromboplastin time; AUC: area under the receiver operating characteristic curve; Baso-%: basophil %; CK: creatine kinase; Cl: chloride; Cre: creatinine; CRP: C-reactive protein; eGFR-Cre: estimated glomerular filtration rate-creatinine; Eosino-%: eosinophil %; GLU: blood glucose; LDH: lactate dehydrogenase; Lympho-Count: lymphocyte count; MCH: mean corpuscular hemoglobin; MCHC: mean corpuscular hemoglobin concentration; MCV: mean corpuscular volume; Mono-Count: monocyte count; MPV: mean platelet volume; N/A: not available; PDW: platelet distribution width; Plt: platelet; PT: prothrombin time; Neutro-%: neutrophil %; RDW: red cell distribution width; SHAP: Shapley Additive Explanations; T-Bil: total bilirubin; TP: total protein; UA: uric acid; UN: urea nitrogen; WBC: white blood cell; γ-GTP: gamma-glutamyl transpeptidase.

To evaluate the predictability of COVID-19 prognosis at initial diagnosis, we constructed prognostic models based on the following information available at that time: symptoms, background information, and biomarkers. Using the severity scale defined by Grein et al [14], we assigned a score to each patient at initial presentation and, if hospitalized, at subsequent time points. The highest score observed across these time points was used as the outcome to be predicted. The AUCs represent the performance of the model in predicting whether patients would reach each severity threshold during hospitalization: 0.717 for severity ≥2, 0.878 for ≥3, 0.951 for ≥4, 0.952 for ≥5, and 0.970 for 6 (ie, death), as shown in Figure 2A. When we examined the concordance between the predicted probability and the actual severity outcome, we found that most patients with an actual severity of ≥3 had a high predicted probability of severity of ≥2. By contrast, patients with an actual severity of 1 or 2 showed a wide distribution of predicted probabilities—from low to high—indicating no clear distinction between the 2 (Figure 2B, top panel). For the other predictions, the predicted probabilities and actual severity showed good concordance; however, some cases remained where the severity risk was not accurately determined (eg, patients who ultimately had mild disease but were predicted to have a high risk of severe illness; Figure 2B).

Subsequently, we evaluated the variable importance of each prediction. The presence of pneumonia was identified as the most important predictor for whether severity would be ≥2 (Figure 2C, leftmost panel). Most hospitalized patients with a severity of ≥2 showed pneumonia at initial diagnosis, and all patients with a severity of ≥3 had pneumonia (Figure 2D). Conversely, as the target severity level of prediction increased, the importance of symptoms decreased. Instead, biomarkers such as the lymphocyte count, prothrombin time (PT), C-reactive protein (CRP), creatinine, and amylase levels emerged as the top predictors (Figure 2C). BMI was also an important factor in predicting whether the severity was ≥4 or ≥5. Age remained an important factor across all severity levels. Among the important predictors, we examined the distribution of age and biomarkers according to actual severity prognosis. Age and BMI were higher in patients with a severity of ≥3 than in others, while biomarkers such as PT, CRP, creatinine, red blood cell volume distribution width, and blood glucose values were higher in patients with a severity of ≥4 (Figure 2E). Lymphocyte counts, estimated glomerular filtration rate (eGFR)-creatinine, and albumin values were lower in more severe cases than in less severe cases, and platelet counts were particularly low in mortality cases (Figure 2E). Amylase levels showed a peculiar distribution: they were low in patients with severity levels 4, 5, and 6, but extremely high in some patients with a severity level of 6 (Figure 2E).

### Dynamic Mortality Risk Assessment Based on Longitudinal Data During Hospitalization

**Figure 3.**
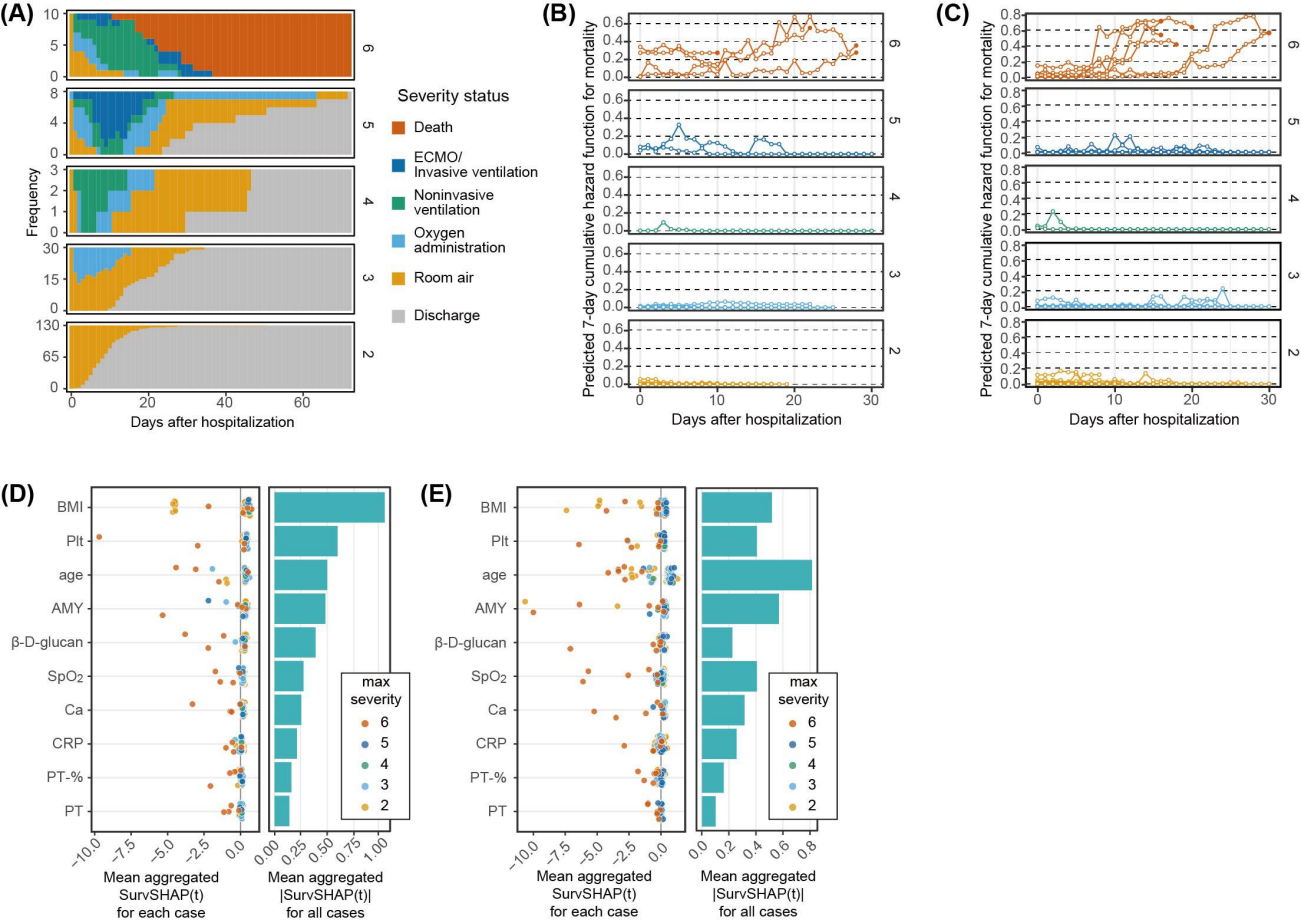
Dynamic mortality risk assessment during hospitalization. (A) Changes in severity status during hospitalization for each severity outcome group. The plot shows how the number of patients receiving procedures such as ventilation or oxygen administration changes over time after admission. (B) Daily mortality risk trajectories for each patient for each day of hospitalization in the test dataset. Mortality risk is shown as the 7-day CHF (ie, the risk of death within 7 days), calculated using the RSF. Filled circles in mortality cases indicate the date of last observation. (C) Daily mortality risk trajectories for each patient for each day of hospitalization in the training dataset. (D) Important variables contributing to the RSF-based mortality prediction. SurvSHAP(t) provides a time-dependent explanation of how each variable influences each patient’s CHF. The left panel shows the mean aggregated SurvSHAP(t) for each case, that is, the mean value of aggregated SurvSHAP(t) across all days of hospitalization. Each circle represents the contribution of a variable to the prediction for each patient in the test dataset. The right panel shows the mean absolute value of SurvSHAP(t) across all patients in the test dataset. (E) Mean aggregated SurvSHAP(t) for each case (left), and mean aggregated |SurvSHAP(t)| across all cases (right) in the training dataset. AMY: amylase; Ca: calcium; CHF: cumulative hazard function; CRP: C-reactive protein; Plt: platelets; PT: prothrombin time; RSF: random survival forests; SpO_2_: peripheral oxygen saturation

Outcome screening based on information from the initial diagnosis was accurate but incomplete. This suggests that a patient’s prognosis is not fully determined at the time of diagnosis and may change depending on the disease course and treatment after admission. When we investigated changes in severity status after admission, we found that oxygen administration and noninvasive ventilation were typically initiated within 5 days after admission, whereas invasive ventilation was often introduced more than 5 days after admission; death generally occurred more than 20 days after admission (Figure 3A). Additionally, many deaths occurred without invasive ventilation (Figure 3A, top panel). This is likely because most mortality cases involved older adults who were not eligible for invasive ventilation or ECMO, even when their condition deteriorated. These observations suggest that, during the weeks between admission and death or discharge, patients undergo changes in their condition that cannot be fully assessed based on oxygen-support status.

We then used survival models, including RSF [19], to evaluate mortality risk during hospitalization. Initially, we attempted to develop a Cox proportional hazards model using all 72 variables, but the model could not be created due to multicollinearity. Therefore, we developed Cox proportional hazards models with L1 (LASSO), L2 (Ridge), and combined L1+L2 (Elastic Net) penalties. The Cox model achieved the highest performance, as evaluated by the C-index, when the Elastic Net penalty was applied. Its performance was comparable to that of the RSF model using all 72 variables (Table 2). When we retrained an RSF model using the top 10 important variables selected during the RSF learning process, as described in the “Methods” section, the performance improved substantially. We also constructed a Cox model based on the same top 10 variables selected by RSF. Although this model exhibited markedly better performance compared with the regularized Cox model, it did not match the predictive accuracy of the RSF model using the same variables. These findings highlight the critical importance of variable selection in enhancing model performance, as well as the value of accounting for interactions and nonlinear effects—factors that the Cox model does not incorporate.

For all 4 mortality cases in the test dataset, an increase in mortality risk was observed 1‐2 weeks before the outcome, and the CHF (ie, the estimated probability of death within 7 days) reached approximately 0.3 or higher at the time of death (Figure 3B). Conversely, in patients recovering from invasive or noninvasive ventilation, the CHF also increased around 1 week after admission, similar to the mortality cases, but then decreased and rarely exceeded 0.2. In mild cases that progressed with oxygen administration or room air, there was little increase in CHF. Similar changes in CHF were observed in the training dataset, which included 6 deaths, suggesting consistency of the predictive model between the training and test datasets (Figure 3C).

The average contribution of the variables to the RSF prediction for each patient was evaluated using the mean aggregated SurvSHAP(t). In addition to background factors such as age and BMI, blood test variables measured multiple times during hospitalization, such as platelets, amylase, and β-D-glucan, had the highest importance in mortality prediction (Figure 3D). Age, amylase level, and platelet count were identified as important predictors of mortality at initial diagnosis. Most of the important variables showed high contributions only in mortality cases; however, some, such as age and BMI, showed a high mean aggregated SurvSHAP(t) even in milder cases, suggesting that they are nonspecific factors. Conversely, important blood test variables such as β-D-glucan, platelets, and calcium (Ca) contributed less in some mortality cases, suggesting heterogeneity of deterioration. In the training data, these predictors also showed high contributions, especially in mortality cases, although there was some shuffling in their rankings (Figure 3E).

**Table 2.** Performance metrics of mortality risk prediction models.

Model	Concordance index	Integrated Brier score	Cumulative area under the receiver operating characteristic curve
Cox-LASSO^a^	0.715	1.14	0.560
Cox-Ridge	0.595	1.53	0.481
Cox ElasticNet	0.845	1.31	0.731
Random survival forests with all 72 variables	0.843	0.664	0.753
Random survival forests with the top 10 variables	*0.941* ^b^	0.315	*0.936*
Cox with the top 10 variables	0.919	*0.272*	0.882

a LASSO: least absolute shrinkage and selection operator.

b Italicized values indicate the model with the best performance for each indicator.

We chose the 7-day time frame because it is clinically meaningful in the context of inpatient care, where monitoring and interventions typically follow a weekly cycle. It provides a practical horizon for anticipating deterioration and making timely care decisions. In addition, the 7-day window offered a suitable balance between the number of outcome events and prediction stability during model development. We also evaluated the data over different periods (eg, 1- and 14-day windows). Although the absolute values of the CHFs differed among the 1-, 7-, and 14-day prediction windows, the temporal patterns within each severity group were approximately consistent (see Figure S2 in Multimedia Appendix 3).

### Rationale for Estimated Mortality Risk in Severe Cases

**Figure 4.**
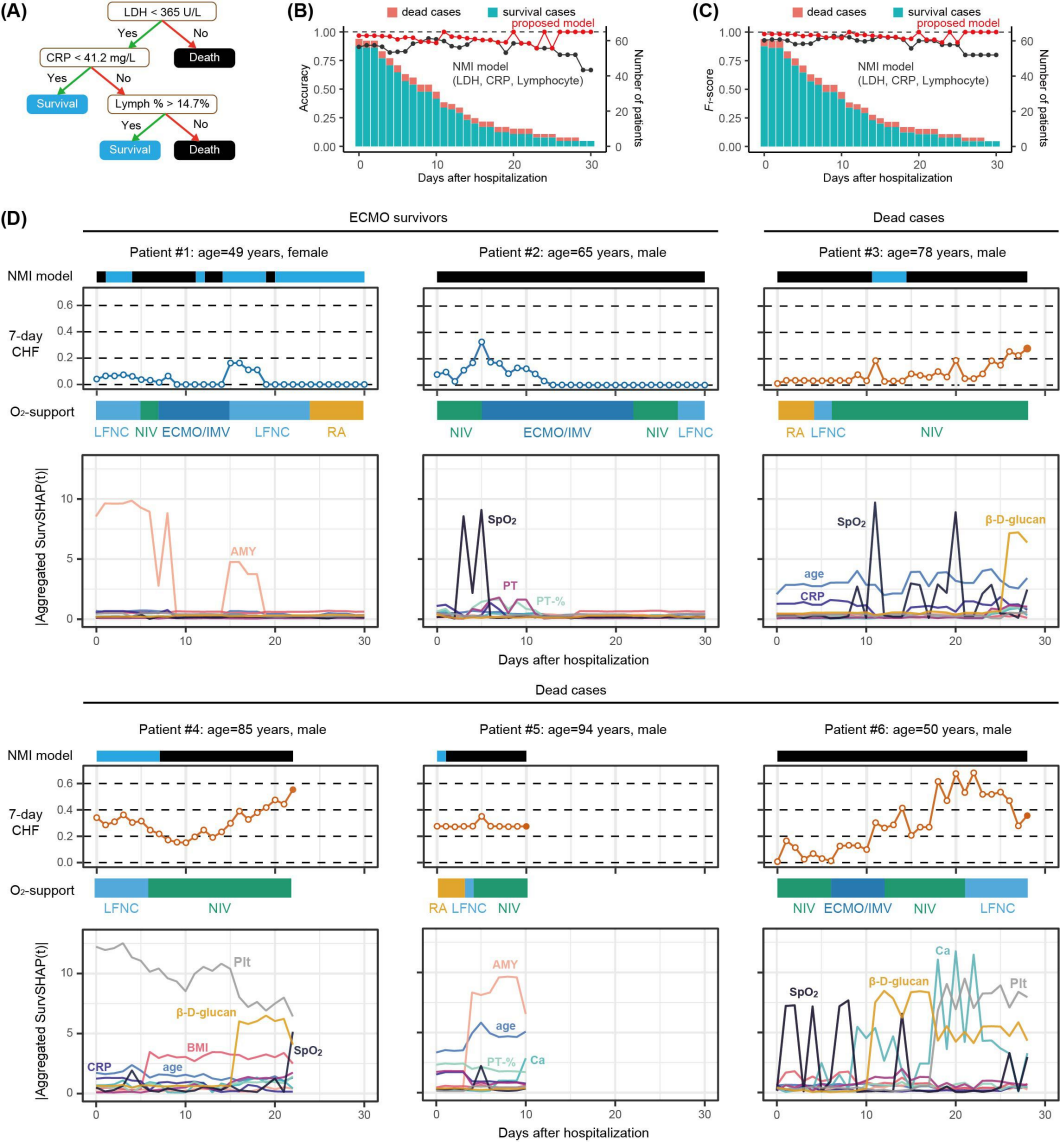
Validation of the RSF model for mortality risk assessment. (A) Mortality risk assessment for COVID-19 based on 3 key predictors identified in a previous study: LDH, lymphocytes, and CRP. (B, C) Comparison of the performance of the RSF model developed in this study with the previously proposed NMI model. Accuracy (B) and *F*_1_-score (C) for distinguishing survival and death cases are shown for each day after hospitalization, based on 61 COVID-19 cases in the test dataset. (D) Changes in the RSF-predicted mortality risk during hospitalization, along with daily SurvSHAP(t) of important predictors for severely ill patients in the test dataset. Six representative severe cases are shown, including 2 ECMO survivors and 4 deaths. The daily SurvSHAP(t) values for each variable correspond to changes in RSF mortality risk. The oxygen-support status is displayed between the CHF plot and the aggregated SurvSHAP(t) plot for each variable, as in Figure 3A: room air is shown in yellow, oxygen administration in light blue, NIV in green, and IMV/ECMO in blue. The light blue and black bars at the top of the graph indicate predictions by the previous NMI model, with light blue for predicted survival and black for predicted death. AMY: amylase; Ca: calcium; CHF: cumulative hazard function; CRP: C-reactive protein; ECMO: extracorporeal membrane oxygenation; IMV: invasive mechanical ventilation; LDH: lactate dehydrogenase; LFNC: low-flow nasal cannula; NIV: noninvasive ventilation; NMI: Nature Machine Intelligence; Plt: platelet; PT: prothrombin time; RA: room air; RSF: random survival forests; SpO_2_: peripheral oxygen saturation.

For the mortality risk assessment of patients with COVID-19, a machine learning model based on blood markers was proposed in Nature Machine Intelligence (NMI) 2020 [21]. This model was built using samples obtained immediately before death or discharge, and lactate dehydrogenase, lymphocytes, and CRP were identified as key features. Mortality risk assessment using a decision tree using these 3 features was proposed in the study to facilitate clinical application (Figure 4A). After approximately 10 days of hospitalization, the RSF and NMI models showed equivalent performance as measured by accuracy and *F*_1_-score. However, immediately following hospitalization, the RSF model outperformed the NMI model, indicating its superiority in early prognostic prediction (Figure 4B and C).

To examine the factors contributing to mortality risk over time, we calculated SurvSHAP(t) daily for the severe cases in the test dataset. Patients and time points showed different combinations of factors associated with changes in CHF (Figure 4D). For example, in young patients #2 and #6, peripheral oxygen saturation (SpO_2_) was associated with an increase in CHF immediately after admission. However, the contribution of SpO_2_ decreased following the initiation of ECMO or invasive ventilation. By contrast, in patients #3 and #4, who were both older and not eligible for ECMO or invasive ventilation, the contribution of SpO_2_ was higher than 10 days after hospitalization. β-D-glucan showed a greater contribution to mortality risk 1‐2 weeks before death than at any other time in patients #3, #4, and #6. Platelets had a high contribution in patients #4 and #6, and BMI also contributed to the mortality risk in these patients. Of note, in patient #4, BMI was measured for the first time on day 7, leading to a rapid increase in its contribution on that day. Calcium and blood amylase levels were elevated immediately before death, suggesting electrolyte abnormalities and multiorgan failure. CRP, a major predictor of mortality at initial diagnosis, contributed to risk assessment immediately after hospitalization.

## Discussion

### Principal Findings

In this study, we developed a framework to assess the prognostic risks of individual patients with COVID-19. Although we were able to predict patient prognosis with reasonable accuracy based on symptoms and blood test results at initial diagnosis, the predicted risk and actual prognosis deviated for some patients, suggesting that prognostic risk may change during hospitalization. Our proposed RSF model demonstrated high performance in predicting in-hospital mortality as a binary outcome, with an *F*_1_-score exceeding 0.95 based on daily predictions made immediately after hospitalization (threshold: CHF>0.2). Visualization of predictors using SurvSHAP(t) made the model explainable for each patient, with consistent predictors observed in both the training and test datasets. Such a dynamic prognostic framework can provide health care providers with real-time guidance to support clinical decision-making during hospitalization.

In the prognostic model at initial diagnosis, we did not predefine a single severity cut-off to dichotomize the outcome. Instead, we explored multiple binary classification tasks based on different thresholds (eg, severity≥1, ≥2,..., ≥5) derived from the original 7-point ordinal outcome scale (0‐6). This approach was chosen to comprehensively assess how predictive performance varies across different levels of clinical severity and to allow for flexible interpretation depending on the clinical context of interest. The partial predictability of disease progression at the time of initial diagnosis can be explained by key variables. CRP levels were important for identifying moderate and severe cases that may require oxygen administration. There was a clear difference in CRP levels between severity levels 3 and 4, but little difference between severity levels ≥4 (Figure 2E). The same was observed for eGFR-creatinine and lymphocyte values, with clear distinctions between severity levels 3 and 4, but minimal differences at higher severities. This suggests that inflammation, reflected by CRP and lymphocytes, and renal dysfunction, reflected by eGFR-creatinine, serve as entry points for severe disease; however, further progression in severity and mortality is mainly influenced by other factors. These findings are consistent with previous studies reporting that elevated CRP and decreased lymphocyte count or eGFR are associated with early COVID-19 severity [22,23]. Our results, however, further suggest that the progression to higher severity and mortality is predominantly driven by additional factors beyond these early indicators. Interestingly, creatinine was not ranked among the top 15 features only in the severity prediction model with the threshold ≥3, while it appeared consistently for other thresholds (Figure 2C). This phenomenon may be attributed to the high degree of information overlap between creatinine and eGFR-creatinine, the latter being a composite measure that incorporates both age and sex and may have provided a more robust signal under this specific threshold. Platelet and amylase levels are the most likely factors associated with the progression of disease severity and mortality, given their importance in prognostic models during hospitalization. Platelet counts were characteristically lower in several mortality cases than in other severe cases, and amylase levels were markedly elevated in some fatal cases (Figure 2E). Fatal cases characterized by these factors may represent patients in whom signs of poor prognosis are already present at initial diagnosis. However, it should be noted that some deaths did not show such signs at initial diagnosis, making them difficult to distinguish from severe or even mild cases.

Although the initial diagnosis model demonstrated fair discriminatory ability overall (AUC 0.717 for predicting severity ≥2), its performance remains below the generally accepted threshold for strong clinical utility (eg, ≥0.8), particularly when applied to low-risk patients. While this AUC is clearly better than random chance (AUC 0.5), it may be insufficient for dependable clinical decision-making when used in isolation. The relatively lower performance in low-risk groups suggests that predicting hospital admission based solely on initial laboratory values may be inherently limited, and that more nuanced clinical judgment—incorporating symptoms, physical findings, and contextual factors—may be necessary in such cases.

Several RSF predictors that varied early in mortality cases were identified. Platelet and PT values were among the top 10 early predictors in the RSF model for COVID-19 mortality. Patients with COVID-19 are prone to arterial and venous thrombosis, and in severe cases, disseminated intravascular coagulation (DIC) can complicate their condition and lead to death [24]. DIC was observed in only 0.6% of COVID-19 survivors, but it occurred frequently in 71.4% of mortality cases [22]. Our results support the idea that coagulation abnormalities, which may lead to DIC, are early predictors of COVID-19 mortality. Amylase was also identified as an important predictor of dynamic mortality risk. It is mainly secreted by the pancreas and salivary glands. Previous studies have reported elevated blood amylase levels in patients with severe COVID-19 [23]. Blood amylase levels are regulated by the balance between amylase production and clearance; elevated levels suggest damage to the producing tissues or impaired kidney function related to clearance. Such tissue damage may be a key event in the death of patients with COVID-19. Changes in blood cell composition were included among the RSF predictors, but these changes were not specific to patients who died (Figure 4). Platelets form platelet-neutrophil complexes when activated, which trigger neutrophil release via neutrophil extracellular traps [25,26]. Neutrophilia is not an independent risk factor for mortality but may act as a predisposing factor for DIC, ultimately leading to death. β-D-glucan is not only an indicator of fungal infection but also a marker of sepsis-related gastrointestinal leakage [27]. Even in the absence of fungemia or bacteremia, blood β-D-glucan levels during COVID-19 infection can induce neutrophil extracellular traps and may be associated with hypercytokinemia and severe inflammation [28]. By integrating these dynamically changing risk factors using RSF, we were able to accurately assess how close each patient was to death at each time point.

Previous studies have used the approach of building models based on data immediately before outcomes such as death or hospital discharge, and then applying these models to earlier time points to predict prognosis [21]. Although this approach can identify factors directly associated with mortality and achieves good predictive accuracy near the outcome, it has not performed well for early prognostic prediction. Even among patients with COVID-19, at least 10 factors are associated with death, and their importance shifts over time: CRP and SpO_2_ are more influential in the earlier phases, whereas β-D-glucan, platelets, and calcium become more important just before death. In patients with COVID-19, viral proliferation is considered the primary pathogenic mechanism during the first few days after onset, whereas the inflammatory response and coagulation abnormalities due to host immunity become the dominant factors after the first week [29]. This shift necessitates early administration of antiviral drugs and neutralizing antibodies during the initial phase, and the use of steroids and anticoagulation therapy after the first week [30,31]. For effective clinical decision-making based on the phase of hospitalization, it is essential to present mortality risk with distinct, phase-specific rationales.

Our prognostic framework can be applied to various aspects of disease prevention and treatment. Early prognostic prediction immediately after hospitalization, which screens for potentially severe cases with high sensitivity, can be used to estimate the number of hospital beds and the amount of medical equipment required. Additionally, owing to the recent shortage of hospital beds, the number of patients receiving treatment at home is increasing, and cases of sudden deterioration or death during home care have become a cause for concern. By using the RSF model to identify early signs of mortality risk, it may be possible to reduce deaths during home treatment and ensure safer management outside the hospital. Coagulation factors, which are key predictors in the RSF mortality model, represent potent targets for early intervention. Heparin administration has been reported to improve the prognosis of severe DIC in patients with COVID-19 [32]. The RSF model may help determine the appropriate timing for initiating anticoagulation therapy and evaluate treatment effects over time.

Although our study utilized a retrospective dataset, we designed the model to simulate real-time clinical application by dynamically updating the CHF at each time point during hospitalization (eg, day t, t+1, t+2). At each time point, only clinical data available up to that day were used, thereby emulating real-world scenarios in which patient information accumulates sequentially. The RSF model recalculates risk scores daily, enabling the timely identification of clinical deterioration and supporting dynamic decision-making. This time-updating mechanism reflects the evolving clinical status of patients and is essential for informing appropriate interventions and care adjustments during hospitalization.

While our framework was developed using data from patients with COVID-19, the underlying approach—time-varying survival modeling with interpretable outputs—is applicable to other acute or chronic diseases characterized by dynamic clinical trajectories. However, we acknowledge that this generalizability has not yet been empirically validated. Future work should explore its application to other conditions, such as sepsis, pneumonia, or heart failure, using disease-specific datasets to evaluate the framework’s adaptability and robustness. To facilitate clinical integration, the model outputs could be embedded into electronic health record systems as real-time dashboards that display individualized risk trajectories and highlight contributing variables. For example, dynamic risk scores and SurvSHAP(t)-based feature summaries could be visualized alongside vital signs or laboratory trends to support triage decisions, early warnings, or escalation of therapy. Although we did not conduct a formal usability study, preliminary feedback from a small group of physicians who viewed the SurvSHAP(t) visualizations suggested that the temporal feature contributions enhanced their understanding of the rationale behind changes in risk over time. Future work should include structured surveys or qualitative interviews to assess how explainability tools influence clinician trust, understanding, and adoption in real-world settings.

In the interpretation of SurvSHAP(t) values, negative values indicate that a variable contributes to an increase in the predicted cumulative hazard, corresponding to a higher mortality risk. Conversely, positive values suggest a protective effect by lowering the predicted risk. SHAP-based feature importance, measured using both the mean absolute SHAP values (|SHAP|) and the mean aggregated absolute SurvSHAP(t) values, which average patient-level contributions over time, reflects the expected magnitude of each variable’s influence on the model output across all observations. This provides insight into how sensitive the model’s predictions are to each variable in aggregate and can be considered a model-based global sensitivity analysis method. In other words, a variable with a larger mean (aggregated) absolute SHAP value is expected to induce greater changes in the model’s output when perturbed, indicating higher sensitivity to that feature.

As an additional aspect of sensitivity analysis, we examined the impact of different missing data scenarios. Preliminary results showed that, although prediction performance occasionally improved slightly without imputation, SHAP analysis revealed that the model was leveraging whether certain symptom variables were measured, essentially using the pattern of missingness as a predictive case. This indicates that the missingness mechanism was likely not at random and may have been biasedly associated with patient outcomes. To prevent overfitting to such data collection artifacts and ensure that the model relied on clinically meaningful features rather than exploiting missingness as a proxy, we opted to train all final models on imputed data.

We recognize that comorbidities may serve as confounders, influencing both predictor variables and patient outcomes. Although comorbidities were included as input features when available, we did not explicitly adjust for them using causal inference frameworks. Future extensions of this work should incorporate structured comorbidity indices (eg, Charlson or Elixhauser indices) and consider techniques such as inverse probability weighting or propensity score adjustment to reduce bias and enhance causal interpretability.

We designed the model with real-time applicability in mind. To assess its suitability for clinical implementation, we quantified the computation time (Table S3 in Multimedia Appendix 6). In our implementation, the learning and inference times for RSF were approximately 1.89 and 0.25 seconds, respectively. Cox regression required approximately 0.10 seconds for learning and 0.01 seconds for inference, making RSF roughly 20 times slower in both processes. However, the computational times for RSF remain sufficiently short to enable inference in real-world clinical settings and do not present a barrier to real-time clinical application. On the other hand, calculating the contribution of variables to the prediction results using SurvSHAP(t) took approximately 20,000 seconds (ie, over 5 hours) for the test dataset of 828 records. Even when converted to computation time per record, the processing remains substantial and may pose a barrier to its use in actual clinical practice. To support future clinical applications, a more efficient and faster implementation of SurvSHAP(t) is desirable.

To evaluate potential biases in dynamic mortality predictions across different demographic groups, we conducted subgroup analyses based on sex and age categories, as summarized in Table 3. Specifically, we assessed performance in the following subgroups: males only, a younger age group (<65 years), and an older age group (≥75 years). The results indicated a slight decrease in predictive performance among males compared with the overall cohort. By contrast, the younger age group showed a marginal performance improvement, likely reflecting the lower incidence of mortality in this subgroup. Conversely, the older age group exhibited a slight reduction in predictive performance, possibly due to the higher mortality rate in this population. Nonetheless, the performance differences across these subgroups were relatively minor compared with the overall analysis, suggesting that the model does not display substantial bias with respect to sex or age. It should also be noted that our dataset consisted exclusively of Japanese patients, limiting our ability to evaluate predictive fairness across different ethnic groups. We acknowledge this limitation and recognize the need for further validation in more diverse populations.

**Table 3.** Performance of mortality prediction in subgroup analyses.

Subgroup	Concordance index
All	0.941
Males only	0.919
Younger age group (<65 years)	0.957
Older age group (≥75 years)	0.864

This study had several limitations. First, the study cohort was relatively small, and data were obtained from a single hospital. As machine learning model performance and robustness are highly influenced by data size and diversity, our models may be prone to overfitting to this specific cohort. As a result, the identified predictive patterns could lack stability and generalizability when applied to other populations without retraining. Furthermore, the substantial reliance on data imputation to address missing values, particularly for certain biomarkers, introduces an additional layer of potential model instability and overfitting risk, as the models may inadvertently learn patterns from the imputation process itself rather than from true underlying biological signals. Consequently, the model will require updates and rigorous revalidation as the study expands to multiple centers and includes a larger sample size, ideally with efforts to minimize the extent of missing data. Second, we included only patients with COVID-19 diagnosed in 2020. Therefore, it is necessary to evaluate the generalizability of these models, including their applicability to different SARS-CoV-2 variants and the effects of vaccination. Notably, the data used in this study were collected during a period when vaccination was rarely administered, and can thus be considered valuable in representing disease trajectories unaffected by vaccination. Third, the study treated daily records from the same patient as independent data points for training, which presents a technical challenge for RSF. Incorporating the risk estimated on the previous day may enable more accurate predictions.

To advance the clinical utility and generalizability of the model, we plan to conduct a multicenter study involving hospitals with diverse patient populations and care protocols. This will enable retraining and external validation of the model across different clinical settings. Additionally, we aim to prospectively evaluate the model in a clinical pilot study, focusing on real-time integration, clinician usability, and assessment of treatment impact through interventional trials.

### Conclusions

We developed and validated an interpretable 2-model framework to enhance prognostic support for patients with COVID-19. The framework integrates a LightGBM model for predicting disease severity at admission with an RSF model that provides daily mortality risk estimates throughout hospitalization. These complementary models are designed to address distinct clinical needs at different stages of patient care.

Central to our approach is interpretability through SurvSHAP(t), which identified actionable, phase-specific predictors, such as elevated CRP levels at admission, declining SpO_₂_ during progression, and late-stage indicators such as platelet loss accompanied by elevated β-D-glucan. These time-dependent explanations empower clinicians to anticipate and respond to evolving patient conditions with greater precision.

By combining early disease stratification with continuous, explainable mortality prediction, our framework facilitates timely interventions and strategic resource allocation. This demonstrates significant potential to improve clinical decision-making in dynamic care environments and may serve as a foundation for managing future infectious disease outbreaks with heterogeneous trajectories, pending prospective multi-institutional validation.

## Supplementary material

10.2196/65585 Multimedia Appendix 1 Descriptive statistics of 84 variables—including background information, symptoms, and blood and urine test data—of patients with COVID-19 for prognostic screening at initial diagnosis.

10.2196/65585 Multimedia Appendix 2 Descriptive statistics of 72 variables—including background information, vital signs, and blood and urine test data—of patients with COVID-19 for dynamic mortality risk assessment during hospitalization.

10.2196/65585 Multimedia Appendix 3 Receiver operating characteristic curves, confusion matrices, and absolute values of the cumulative hazard functions.

10.2196/65585 Multimedia Appendix 4 Performance of hospitalization prediction models, including Light Gradient Boosting Machine, random forest, support vector machine, Elastic Net, and decision tree.

10.2196/65585 Multimedia Appendix 5 Detailed methods covering missing data imputation, hyperparameter optimization, ensemble averaging, evaluation metrics, model selection and interpretability rationale, and clinical justification of selected predictors.

10.2196/65585 Multimedia Appendix 6 Comparison of computation time for training, prediction, and Shapley Additive Explanations–based interpretation in survival analysis.

## Abbreviations

AUC: area under the receiver operating characteristic curve
C-index: concordance index
CHF: cumulative hazard function
CRP: C-reactive protein
DIC: disseminated intravascular coagulation
ECMO: extracorporeal membrane oxygenation
eGFR: estimated glomerular filtration rate
GBDT: gradient boosting decision trees
LASSO: least absolute shrinkage and selection operator
LightGBM: Light Gradient Boosting Machine
NMI: Nature Machine Intelligence
PT: prothrombin time
RSF: random survival forests
SHAP: Shapley Additive Explanation
SpO_2_: peripheral oxygen saturation
