# Supplementary material for: Explainable Machine Learning Framework for Dynamic Monitoring of Disease Prognostic Risk: Retrospective Cohort Study
Source: JMIR Form Res. 2025 Aug 7;9:e65585. doi: 10.2196/65585 (PMC12501906; doi:10.2196/65585)

Figure S1: (a) ROC curves and (b) confusion matrices for the five machine learning models predicting hospitalization.

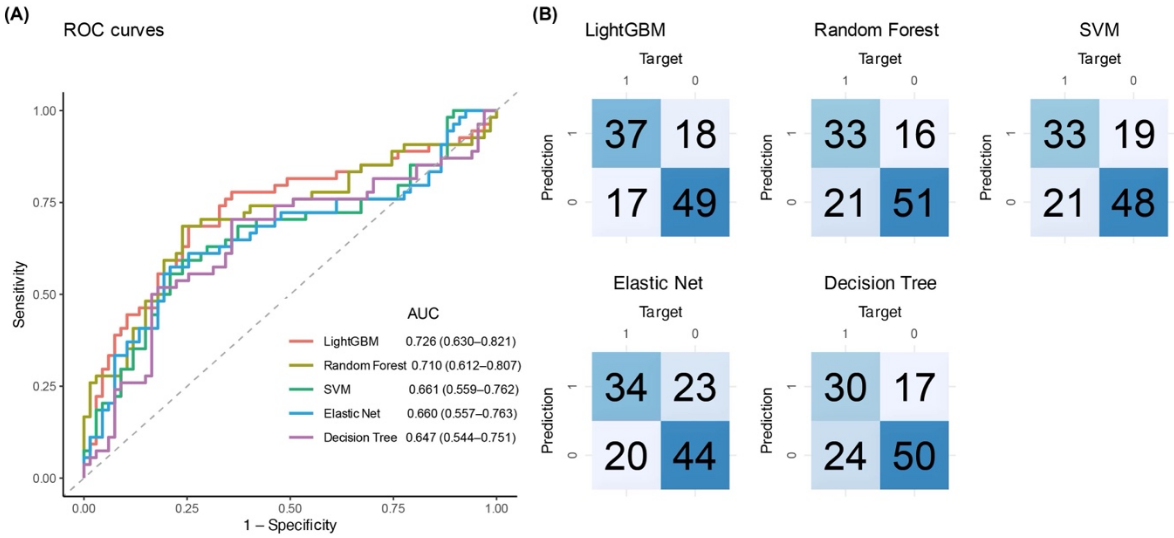

Figure S2: The absolute values of the cumulative hazard functions (CHFs) among the 1-day, 7-day, and 14-day prediction windows.

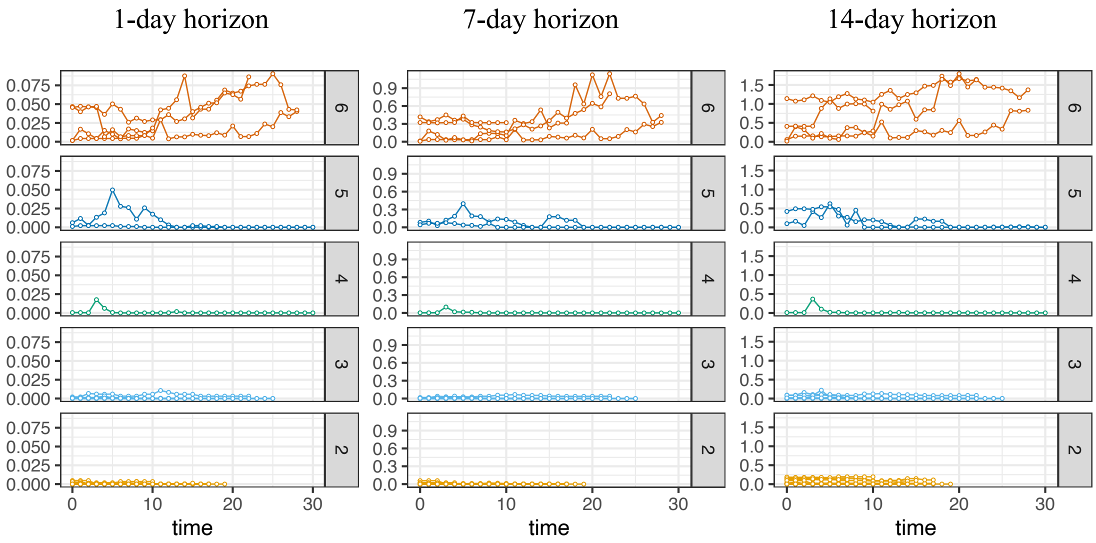

Supplement: Multimedia Appendix 3 [file formative-v9-e65585-s003.pdf]
