# Supplementary material for: Explainable Machine Learning Framework for Dynamic Monitoring of Disease Prognostic Risk: Retrospective Cohort Study
Source: JMIR Form Res. 2025 Aug 7;9:e65585. doi: 10.2196/65585 (PMC12501906; doi:10.2196/65585)
Supplement: Multimedia Appendix 4 [file formative-v9-e65585-s004.pdf]

Multimedia Appendix 4 (Table S3). Evaluation of performance and computational efficiency in machine learning models for hospitalization prediction

Performance of Hospitalization Prediction Models						
Model	Accuracy	Sensitivity	Specificity	PPV[1]	NPV[2]	Time [s][3]
LightGBM	**0.711**	**0.685**	0.731	**0.673**	**0.742**	1,189.8
Random Forest	0.694	0.611	**0.761**	**0.673**	0.708	22,514.0
SVM	0.669	0.611	0.716	0.635	0.696	157.0
Elastic Net	0.645	0.630	0.657	0.596	0.688	141.0
Decision Tree	0.661	0.556	0.746	0.638	0.676	**71.0**

Best value(s) depected in bold.

[1] Positive Predictive Value (PPV)

[2] Negative Predictive Value (NPV)

[3] Computation times include hyperparameter tuning, prediction, and SHAP analysis. Computations were performed on a MacBook Pro (Apple M1, 8 cores, 16 GB RAM, macOS Sonoma 14.4).
