# Supplementary material for: Explainable Machine Learning Framework for Dynamic Monitoring of Disease Prognostic Risk: Retrospective Cohort Study
Source: JMIR Form Res. 2025 Aug 7;9:e65585. doi: 10.2196/65585 (PMC12501906; doi:10.2196/65585)
Supplement: Multimedia Appendix 5 [file formative-v9-e65585-s005.pdf]

Supplementary Methods: Detailed Methods covering missing-data imputation, hyperparameter optimization, ensemble averaging, evaluation metrics, model-selection and interpretability rationales, and clinical justification of selected predictors.

Handling of missing data in the initial prognosis model development

Missing values were imputed using the missForest algorythm, which applies a non-parametric, Random Forest–based method to iteratively predict missing values and capture nonlinear relationships among variables.

Although missing values in the training data were imputed before learning to reduce bias, prediction was performed for the test data containing missing values. This is because, while LightGBM can handle missing values during prediction, the presence or absence of a value itself may carry predictive information. For instance, certain features may only be measured for more severe cases, suggesting that the missingness is not completely at random (MCAR), but instead may follow a missing at random (MAR) or even not at random (MNAR) mechanism.

Nevertheless, to reduce potential bias during model training, we imputed missing values in the training data using a machine learning-based multivariate imputation approach. This method

leveraged the correlations among features to estimate missing values from the observed values of other features.

Handling of missing data in the survival model development

The missing values were imputed using the most recent values until the next values were measured, i.e., the last observation carried forward method. If there were no values before imputation, the median values for numerical values and mode values for categorical values measured for all patients were applied. For BMI, height, and weight, missing values with no previous measurements were imputed as the median values for men and women.

Hyperparameter tuning and model ensembling for LightGBM

To build the LightGBM model during the training phase, we performed hyperparameter tuning by Bayesian optimization with repeated cross-validation on the training dataset using the Optuna version 4.2.1.

To address class imbalance, we fixed class_weight="balanced" and tuned the following hyperparameters, aiming to maximize average_precision:

max_depth (integer range: 2–10),

min_child_samples (integer range: 5–100),

num_leaves (integer range: 2–256),

feature_fraction (float range: 0.4–1.0),

bagging_fraction (float range: 0.4–1.0),

bagging_freq (integer range: 1–7),

lambda_l1 (float range: 1e-8–10.0), and

lambda_l2 (float range: 1e-8–10.0).

The hyperparameter tuning was performed using optuna.integration.OptunaSearchCV, where n_trials was set to 10 and cross-validation was carried out with RepeatedStratifiedKFold(n_splits=5, n_repeats=10) on the training data. Using the optimal hyperparameters identified through this process, we then retrained (refit) the model on the entire training dataset, thereby creating a predictive model. This entire optimization procedure was then repeated 100 times with different random_seed values, and the resulting 100 models were averaged to form an ensemble designated as the best model.

Hyperparameter setting for Random Survival Forest and regularized Cox models

For the construction of the RSF models, we adopted parameter settings that have consistently demonstrated good performance across various types of electronic health record (EHR) data based on our prior experience. Specifically, we set

n_estimators=2000,

min_samples_split=10,

min_samples_leaf=15, and

max_features="sqrt".

The regularization strength (α) for each regularized Cox model was optimized through grid search using the concordance index (C-index) as the evaluation metric.

Evaluation metrics for survival models

- **Concordance Index (C-index):** The C-index is defined as the proportion of comparable case pairs in which the predicted risk scores correctly rank the order of event times. Two cases are considered comparable when the event occurred in the one with the shorter observed time.
- **Integrated Brier Score (IBS):** The time-dependent Brier score extends the mean squared error to right-censored data, assessing the accuracy of predicted probabilities relative to actual outcomes. The IBS is computed as the integral of the time-dependent Brier score over a specified time interval, weighted by the time-to-event distribution. In this study, we used a time interval from 1 to 50 days after the observation.
- **Mean Cumulative/Dynamic Area Under the ROC Curve (Mean AUC):** The cumulative/dynamic AUC is a time-dependent extension of the traditional AUC, reflecting sensitivity and specificity across time. The mean AUC is calculated as the integral of the cumulative/dynamic AUC over the specified interval, weighted by the Kaplan–Meier estimator. The same time interval as for the IBS (1 to 50 days) was used.

Rationale for selecting Random Survival Forests

Although deep-learning survival models, such as Dynamic-DeepHit [1] and DySurv [2] can also capture complex temporal patterns, they typically require larger sample sizes and substantial GPU resources for training and inference, and their attribution methods for interpretability often remain experimental. In contrast, Random Survival Forests (RSF) strike a pragmatic balance. RSF models natively handle mixed data types, can achieve competitive accuracy even in moderately sized cohorts (such as the <500 cases in our study), and their learning and inference

processes can be efficiently performed on standard CPUs without the need for high-performance computational resources like GPUs. Crucially, the tree-based structure of RSF enables the use of methods like SurvSHAP(t) to deliver patient-specific, time-resolved explanations, a capability that is not yet as mature or readily available for neural survival architectures.

Rationale for selecting SurvSHAP(t) for model interpretation

While Random Survival Forests (RSF) are inherently more interpretable than many "black-box" models, deriving detailed, patient-specific, and time-dependent explanations requires specialized techniques. Several methods can provide insights into RSF predictions, but many possess limitations regarding the nuanced understanding required in dynamic clinical risk assessment. Commonly, global feature importance measures, such as Permutation Importance or minimal-depth statistics [3], are used with tree-based ensembles. While useful for identifying overall influential predictors, these methods provide a single importance score per feature across the entire dataset. They do not explain why a specific prediction was made for an individual patient, nor do they inherently capture how a feature's importance might change over the course of an illness (i.e., they are not time-dependent).

In contrast, and crucially for dynamic prognostic models, SurvSHAP(t) is designed to explain predictions from survival models at different time points. This allows us to understand how the importance of various clinical variables (e.g., CRP, SpO2) evolves throughout a patient's hospitalization, directly addressing our objective of tracking changing risk drivers.

In addition, SHAP values ensure local accuracy (the sum of feature attributions equals the difference between the prediction and the average prediction) and consistency (a feature's contribution does not counterintuitively change if the model changes to rely more on that feature). This provides more reliable insights into the model's decision-making process.

Therefore, SurvSHAP(t) provides a superior approach for our study by offering interpretable, patient-specific, and time-dependent feature attributions that are consistent and directly derived from the RSF model structure. This capability is essential for clinicians to understand not just what a patient's risk is, but why it is at that level and how those driving factors change over time, facilitating more informed clinical decision-making.

Clinical justification for variables selection

The top 10 variables selected by RSF collectively span baseline predisposition (age, BMI), respiratory failure (SpO_2), systemic inflammation (CRP), coagulation dysregulation (platelets, PT-%/PT, calcium), secondary infection or gut leakage (β-D-glucan), and organ injury (amylase). Their convergence captures the multifactorial pathophysiology of COVID-19, providing a clinically coherent and mechanistically diverse feature set rather than a list dictated solely by computational rankings.

Supplementary References

[1] Lee C, Yoon J, Van Der Schaar M. Dynamic-deephit: A deep learning approach for dynamic survival analysis with competing risks based on longitudinal data. IEEE Transactions on Biomedical Engineering. 2019 Apr 3;67(1):122-33.

[2] Mesinovic M, Watkinson P, Zhu T. DySurv: dynamic deep learning model for survival analysis with conditional variational inference. Journal of the American Medical Informatics Association. 2024 Nov 21:ocae271.

[3] Ishwaran H, Kogalur UB, Gorodeski EZ, Minn AJ, Lauer MS. High-dimensional variable selection for survival data. Journal of the American Statistical Association. 2010 Mar 1;105(489):205-17.
