# Supplementary material for: Explainable Machine Learning Framework for Dynamic Monitoring of Disease Prognostic Risk: Retrospective Cohort Study
Source: JMIR Form Res. 2025 Aug 7;9:e65585. doi: 10.2196/65585 (PMC12501906; doi:10.2196/65585)
Supplement: Multimedia Appendix 6 [file formative-v9-e65585-s006.pdf]

Multimedia Appendix 6 (Table S4).
Comparison of computation time for training, prediction, and
Shapley Additive Explanations-based interpretation in survival analysis

Computation time measured in seconds			
Model	Method	#records	Computation time [s][1]
Cox	Training	1736	0.104
	Prediction	828	0.014
RSF with top 10 variables	Training	1736	1.894
	Prediction	828	0.251
	SurvSHAP(t)	828	19783.459

[1] Computations were performed on a workstation equipped with an AMD Ryzen Threadripper PRO 5975WX 32-core CPU, 130 GiB of RAM, running Ubuntu 20.04.5 LTS.
